# A long survivor with local relapse of hilar cholangiocarcinoma after R1 surgery treated with chemoradiotherapy: a case report and literature review

**DOI:** 10.1186/s40792-016-0195-9

**Published:** 2016-07-04

**Authors:** Hirohisa Okabe, Akira Chikamoto, Masataka Maruno, Daisuke Hashimoto, Katsunori Imai, Katsunobu Taki, Kota Arima, Takatoshi Ishiko, Hideaki Uchiyama, Toru Ikegami, Norifumi Harimoto, Shinji Itoh, Tomoharu Yoshizumi, Toru Beppu, Hideo Baba, Yoshihiko Maehara

**Affiliations:** Department of Gastroenterological Surgery, Graduate School of Life Sciences, Kumamoto University, 1-1-1 Honjo, Kumamoto, Kumamoto 860-8556 Japan; Department of Multidisciplinary Treatment for Gastroenterological Cancer, Kumamoto University Hospital, 1-1-1 Honjo, Kumamoto, Kumamoto 860-8556 Japan; Department of Surgery and Science, Graduate School of Medical Sciences, Kyushu University, 1-1-1 Honjo, Kumamoto, Kumamoto 860-8556 Japan

**Keywords:** Hilar cholangiocarcinoma, Radiation, Chemotherapy

## Abstract

The treatment outcome of extrahepatic cholangiocarcinoma remains insufficient because it is difficult to obtain accurate diagnosis of tumor spreading and effective treatment agent is quite limited in spite of substantial current efforts, all of which have been unsuccessful except for gemcitabine plus cisplatin. The patient was a 60-year-old female who had developed hilar cholangiocarcinoma and underwent extrahepatic bile duct resection. Although it was conceivable that it would be the R1 resection, the patient wanted to receive limited resection to avoid postoperative complication mainly because she was depressed. In histology, interstitial spreading of tumor was appreciated at the surgical margin of bile duct. The patient did not accept to receive the additional treatment after the surgery and hardly visited the hospital to take the periodical test for monitoring the residual cancer cells. As expected, the local relapse of tumor was appreciated 1 year after the R1 surgery. She chose radiotherapy and agreed with subsequent S-1 treatment for 26 months. Consequently, elevated CA19-9 was decreased, and local relapse has been successfully controlled for more than 7 years after the relapse of tumor. Here, we report quite a rare case in terms of long survivor after chemoradiotherapy on locally relapsed unresectable hilar cholangiocarcinoma.

## Background

Curative resection of extrahepatic cholangiocarcinoma remains challenging mainly due to the limitation of diagnosis regarding vertical and horizontal tumor spreading. Although prognosis of R1 surgery on extrahepatic cholangiocarcinoma is reported to be better than that of best supportive care, the optimal additional treatment for R1 surgery is controversial and has not been reviewed so far. Here, we aim to report a case who has successfully survived for long time after R1 surgery on hilar cholangiocarcinoma and review the treatment on extrahepatic cholangiocarcinoma patients with R1 surgery.

Surgical resection or liver transplantation remains the only curative treatment that can offer long-term survival for hilar cholangiocarcinoma (Hl-CC) [1, 2]. Despite the preoperative and surgical advances for Hl-CC, some patients still have microscopic positive resection (R1) and recurrence occurs in many of them even after curative resection (R0). For patients who unfortunately have unresectable Hl-CC or the unresectable recurrence after curative resection, gemcitabine plus cisplatin is the only effective therapeutic agent that has been globally appreciated based on the proper clinical study to date [3]. Here, we report the case representing the local relapse of Hl-CC after R1 surgery and long-term survival with the treatment of radiation and subsequent chemotherapy and discuss the previous outcome of concomitant treatment plus surgery in extrahepatic cholangiocarcinoma (ECC) patients with R1 surgery.

## Case presentation

**Fig. 1:**
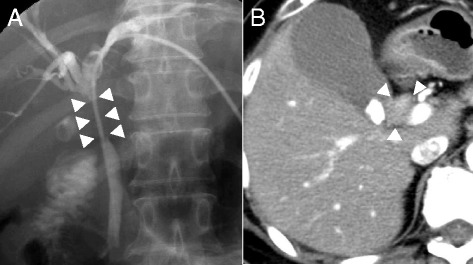
Preoperative imaging of hilar cholangiocarcinoma. **a** Stenosis of proximal biliary tract is appreciated through percutaneous transhepatic biliary drainage catheter. **b** Computed tomography shows the tumor with slight enhancement. *Arrow* represents the tumor

A 60-year-old female visited a local hospital due to jaundice but did not have abdominal pain. She was then referred to our department for the surgical treatment. Total bilirubin level was 25.6 mg/dL, and enzymes, such as AST, ALT, and gamma-GTP, are also high. Percutaneous transhepatic bile duct drainage (PTBD) was performed, and cholangiography was done (Fig. 1a). In contrast enhanced computed tomography (CT), there was an enhanced lesion in hilar biliary tract and dilation of intrahepatic duct was appreciated (Fig. 1b). With the endoscopic retrograde cholangio-pancreatography (ERCP), there was an obstruction of biliary tract and biopsy-proved adenocarcinoma. Hence, she was diagnosed with hilar bile tract carcinoma. There was no distant metastasis. She was depressed, but the disease was under control with the medication.

**Fig. 2:**
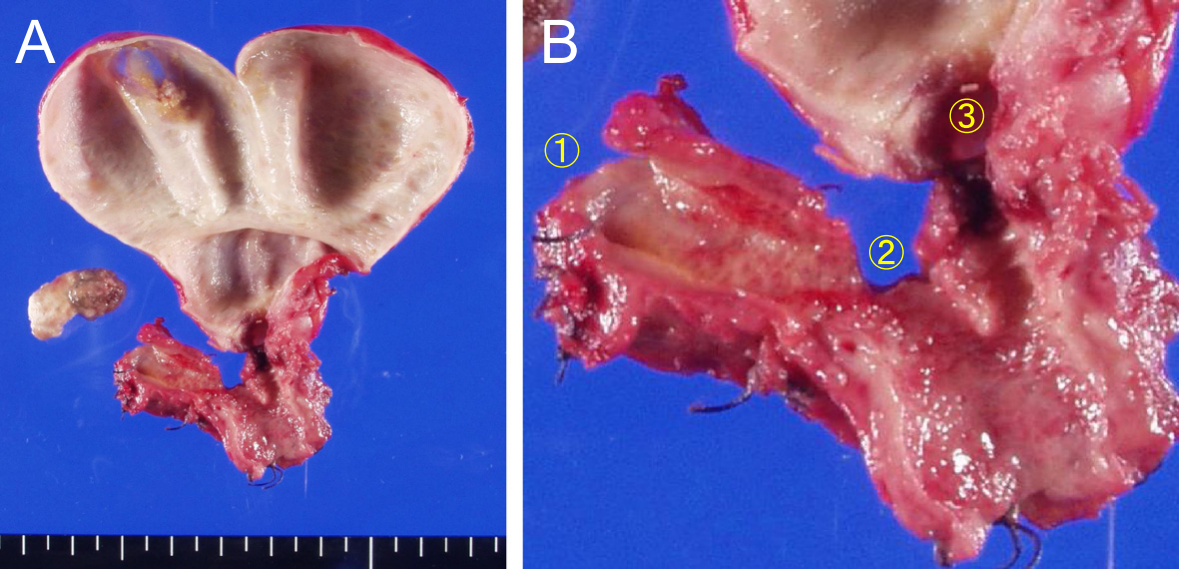
The specimen of hilar cholangiocarcinoma extending to cystic duct. **a** Extrahepatic bile duct was resected. **b** *Circled 1* represents right hepatic duct; *circled 2* represents left hepatic duct; *circled 3* represents cystic duct. Abnormal epithelia is widely seen in resected biliary tract including cystic duct

**Fig. 3:**
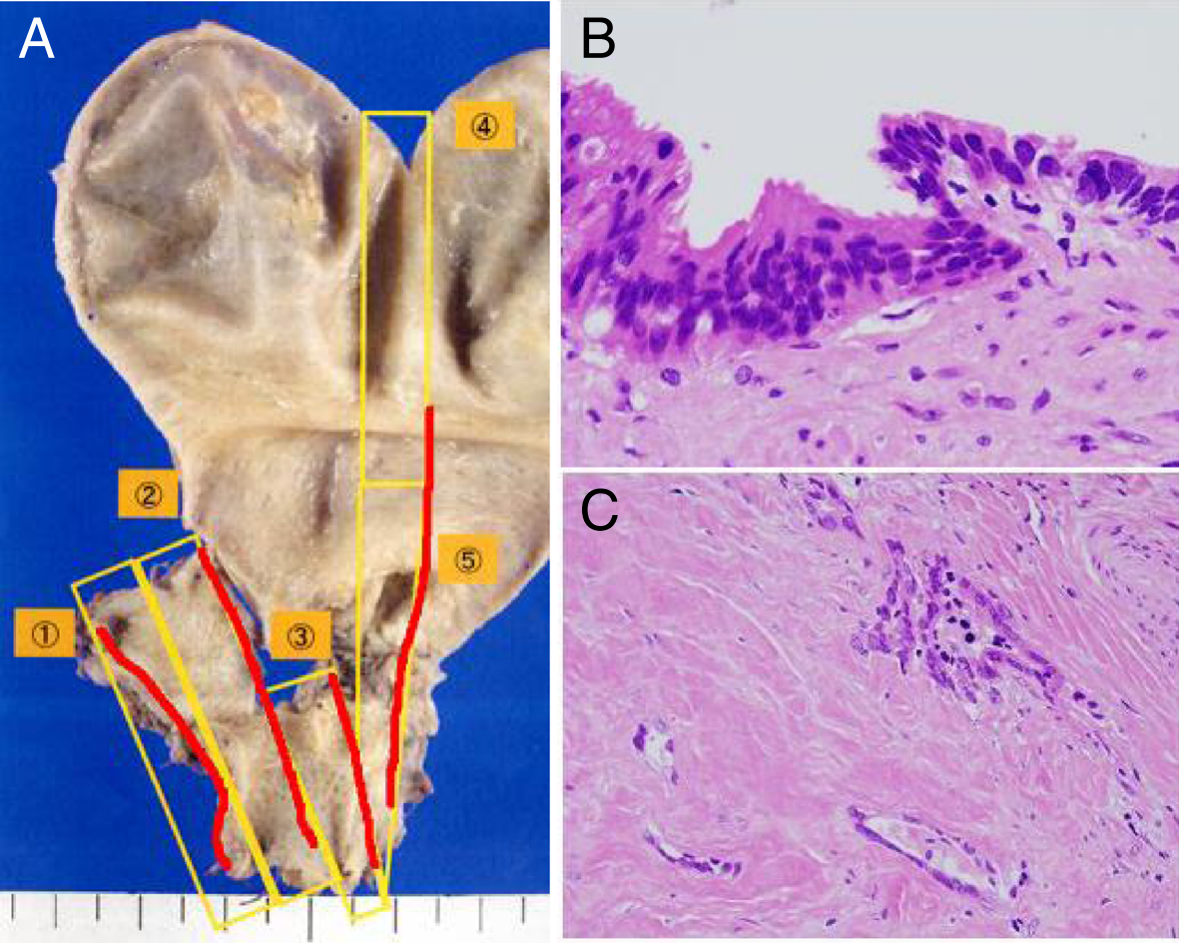
Pathological findings of hilar cholangiocarcinoma. **a** Extension of the hilar cholangiocarcinoma. *Red line* represents the existence of cancer cells evaluated with several paraffin blocks (*open yellow rectangles*). Paraffin block numbers are shown as *orange-colored circled numbers*. **b** Cholangiocarcinoma cells are seen in biliary epithelia at the surgical margin on hepatic side. **c** Cholangiocarcinoma cells are also seen in submucosal area. ×400 magnification

After the jaundice improved, we planned right hemihepatectomy and extrahepatic bile duct resection. However, the patient rejected extensive surgery. The reason was that she wanted to have postoperative complications not to worsen her depressive condition. Finally, she chose limited operation which was the resection of extrahepatic bile duct (Fig. 2a). Gall stone was appreciated and wall thickness was extended into the gall bladder (Fig. 1b). Extension of the lesion (red line) is shown in Fig. 3a. The lesion expanded into the gall bladder. The tumor was well-differentiated adenocarcinoma with no lymph node metastasis. Perineural invasion was positive, and lymphovascular invasion was negative. Surgical margins at both proximal (hepatic) and distal (pancreatic) side were positive. Carcinoma cells were appreciated at both biliary epithelia (Fig. 3b) and submucosal stroma (Fig. 3c).

**Fig. 4:**
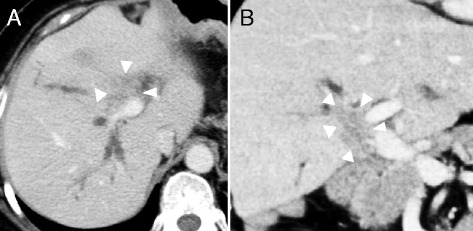
Computed tomography of the relapse of cholangiocarcinoma. **a**, **b** Intrahepatic bile duct is dilated, and low-density tumor is appreciated at the hilum of the liver. *Arrows* represent the low density tumor which is the relapse of residual cancer cells at surgical margin of biliary tract

**Fig. 5:**
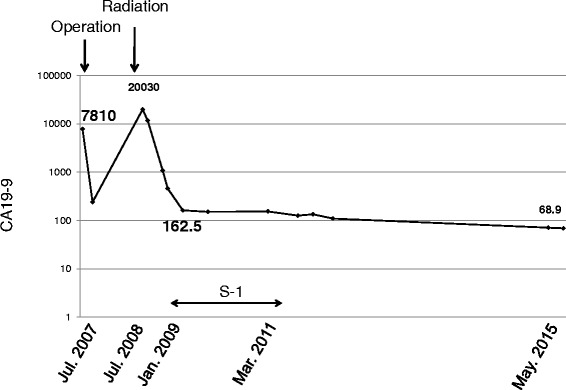
Change of CA19-9 level. CA19-9 level decreased after surgery and re-elevated at the tumor relapse. After the radiation and subsequent chemotherapy, the level was decreased and controlled for more than 6 years

We recommended her to receive chemotherapy, but she declined to do it. In addition, she hardly came to the hospital for the routine test. One year later, in contrast enhanced CT, intrahepatic bile duct was dilated (Fig. 4a) and abnormal thick soft tissue at anastomotic site showed up (Fig. 4b). Tumor marker CA19-9 was remarkably elevated (Fig. 5). Local relapse of residual cancer cells was macroscopically appreciated as an inevitable consequence of R1 surgery. Since standard chemotherapy was not yet established at that time, she chose the radiotherapy. The patient underwent CT simulation, and clinical target volume was the gross total volume which was the recurrent mass seen on CT plus 0.5–1.0-cm margin. Three-dimensional comformal radiotherapy was planned, and the clinical target volume was irradiated with 50 Gy at a daily dose of 2.0 Gy. After 50 Gy of radiation was completed, the dilation of biliary duct was improved. In addition, we recommended her to subsequently receive chemotherapy with tegafur-gimeracil-oteracil-potassium (S-1, 100 mg/body). Since she was tolerable to keep taking the drug, S-1 treatment was continued for 26 months. There has been neither re-elevation of CA19-9 nor relapse of tumor for more than 7 years since S-1 treatment was stopped.

### Discussion

**Fig. 6:**
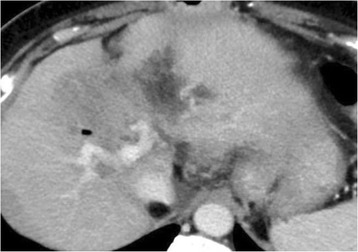
Computed tomography of the treated liver 6 years after the tumor relapse. Neither dilatation of intrahepatic bile duct nor tumor relapse is seen in the latest CT imaging

The patient who has successfully controlled the local relapse of Hl-CC after R1 surgery is introduced in the current study. To the best of our knowledge, this is quite a rare case because patients who have interstitial invasion of cancer cells at the surgical margin show worse prognosis than superficial spreading of cancer cell [4, 5], suggesting that additional locoregional treatment strategy might improve the elimination of microscopic residual cancer cells (Fig. 6). A review of the literature allowed the identification of several reports proposing additional treatment on R1 patients (Table 1). Large scale study in Korea showed that R1 surgery plus radiotherapy or chemoradiotherapy improved the occurrence of local recurrence rate and that both concomitant chemotherapy and chemoradiotherapy improved the occurrence of distant metastasis [6]. Although the benefit of concomitant chemotherapy needs further investigation, local relapse rate shown in several studies might decrease by additional radiotherapy with or without chemotherapy; 12–39 % versus surgery alone or surgery plus chemotherapy; 58–59 % [6–9]. However, local relapse also depends on the biological characteristics and interstitial spreading of cancer cell [4]. Population-based analysis from the University of Texas suggested that adjuvant radiotherapy improved early survival compared with surgery alone in a multifactorial model, although adjuvant RT may be associated with long-term (>5 years) survival decrement in univariate analysis [10].

**Table 1:** Outcome of concomitant treatment plus R1 surgery in patients with ECC

Year	Total number	Patient and treatment for residual cancer cells	Number of R1 patients	Locoregional failure (%)	*P* value	Distant failure (%)	*P* value	Ref
2006	81	Surgery + RT (45–59 Gy) + CTx (5FU^a^ or GEM)	R0:12, R1:16	11 (39.3)	–	7 (25.0)	–	9
2012	25	Surgery + RT (45–50Gy) + CTx (GEM)	8	1 (12.5)	–	4 (50.0)	–	8
2013	25	Surgery → local relapse → EBRT (48Gy) + CTx (CDDP or 5FU^a^)	6	2 (33.3)	–	3 (50.0)	–	7
2015	336	Surgery alone	22	13 (59.1)	0.003	16 (72.7)	0.007	6
		Surgery + CTx (5FU^a^ + CDDP or GEM)	12	7 (58.3)		3 (25.0)		
		Surgery + RT (40–50 Gy)	13	2 (15.4)		6 (46.2)		
		Surgery + CRT (5FU^a^ or GEM + RT)	20	3 (15.0)		5 (25.0)		

*RT* radiation, *CTx* chemotherapy, *GEM* gemzal, *EBRT* external beam radiation therapy, *CDDP* cisplatin, *CRT* chemoradiation

^a^5FU: 5-Fluorouracil-based chemotherapy such as capecitabine, TS1, and infusion of 5FU

To date, only gemcitabine plus cisplatin has been accepted for first-line therapy for unresectable biliary tract cancer [3], and the subsequent challenges fail to establish the next promising therapeutic agent [11, 12]. S1 treatment might be effective for some patients if we can select the good responder to S1 treatment or arrange the optimal dose of drug [13]. In this case, S1 treatment plus radiotherapy was eventually effective. However, it was difficult to determine when adjuvant S1 treatment was finished. The patient hoped to keep the S1 treatment, and we continued it for more than 2 years. Translational research is required for finding the biomarker selecting those patients responsive to 5-FU based treatment or the new strategy of how to use 5-FU based chemotherapeutic agent and how long to continue the treatment. Since the efficacy of radiotherapy alone is limited in terms of the locoregional therapy, the combination of chemotherapy and radiotherapy seems to be best additional option to control residual cancer cells at the locoregional area as well as latent cells in distant organ. Furthermore, postoperative complications postpone the chance of adjuvant chemotherapy resulting in the propagation of residual cancer cells. In this sense, neoadjuvant supportive treatment might be the best way to control microscopic cancer cells both around the main tumor and in distant organ, which are hard to eradicate by surgery alone. Neoadjuvant chemoradiation plus liver transplantation provided promising outcome for patients with perihilar cholangiocarcinoma [14].

## Conclusions

R0 resection is the most effective treatment for hilar cholangiocarcinoma. However, we encounter the patients who resulted in receiving R1 surgery in spite of extended surgical procedure due to the limitation of accuracy in diagnosis. Concomitant treatment for ECC patients with R1 surgery has not been established. Local relapse after R1 surgery was controlled by chemoradiation for long term in the current case. Since survival rate of R1 surgery is worse than R0 surgery in ECC [4], patients with borderline resectable ECC, who are potentially candidates for R1 surgery, need optimal multimodal treatment in addition to the planned surgery.

## Consent

Written informed consent was obtained from the patient for publication of this Case Report and any accompanying images.

## References

[1] Rea DJ, Heimbach JK, Rosen CB, Haddock MG, Alberts SR, Kremers WK (2005). Liver transplantation with neoadjuvant chemoradiation is more effective than resection for hilar cholangiocarcinoma. Ann Surg..

[2] Patel T (2011). Cholangiocarcinoma—controversies and challenges. Nat Rev Gastroenterol Hepatol..

[3] Valle J, Wasan H, Palmer DH, Cunningham D, Anthoney A, Maraveyas A (2010). Cisplatin plus gemcitabine versus gemcitabine for biliary tract cancer. New Engl J Med..

[4] Igami T, Nagino M, Oda K, Nishio H, Ebata T, Yokoyama Y (2009). Clinicopathologic study of cholangiocarcinoma with superficial spread. Ann Surg..

[5] Sakamoto E, Nimura Y, Hayakawa N, Kamiya J, Kondo S, Nagino M (1998). The pattern of infiltration at the proximal border of hilar bile duct carcinoma: a histologic analysis of 62 resected cases. Ann Surg..

[6] Im JH, Seong J, Lee IJ, Park JS, Yoon DS, Kim KS et al. Surgery alone versus surgery followed by chemotherapy and radiotherapy in resected extrahepatic bile duct cancer: treatment outcome analysis of 336 patients. Cancer Res Treat: official journal of Korean Cancer Association. 2015. doi:10.4143/crt.2015.091. 10.4143/crt.2015.091 PMC4843751 26323644

[7] Moureau-Zabotto L, Turrini O, Resbeut M, Raoul JL, Giovannini M, Poizat F (2013). Impact of radiotherapy in the management of locally advanced extrahepatic cholangiocarcinoma. BMC cancer..

[8] Habermehl D, Lindel K, Rieken S, Haase K, Goeppert B, Buchler MW (2012). Chemoradiation in patients with unresectable extrahepatic and hilar cholangiocarcinoma or at high risk for disease recurrence after resection: analysis of treatment efficacy and failure in patients receiving postoperative or primary chemoradiation. Strahlenth Onkol: Organ der Deutschen Rontgengesellschaft.

[9] Ben-David MA, Griffith KA, Abu-Isa E, Lawrence TS, Knol J, Zalupski M (2006). External-beam radiotherapy for localized extrahepatic cholangiocarcinoma. Int J Radiat Oncol Biol Physics..

[10] Fuller CD, Wang SJ, Choi M, Czito BG, Cornell J, Welzel TM (2009). Multimodality therapy for locoregional extrahepatic cholangiocarcinoma: a population-based analysis. Cancer..

[11] Lee J, Park SH, Chang HM, Kim JS, Choi HJ, Lee MA (2012). Gemcitabine and oxaliplatin with or without erlotinib in advanced biliary-tract cancer: a multicentre, open-label, randomised, phase 3 study. Lancet Oncol..

[12] Malka D, Cervera P, Foulon S, Trarbach T, de la Fouchardiere C, Boucher E (2014). Gemcitabine and oxaliplatin with or without cetuximab in advanced biliary-tract cancer (BINGO): a randomised, open-label, non-comparative phase 2 trial. Lancet Oncol..

[13] Saif MW, Choma A, Salamone SJ, Chu E (2009). Pharmacokinetically guided dose adjustment of 5-fluorouracil: a rational approach to improving therapeutic outcomes. J Natl Cancer Inst..

[14] Darwish Murad S, Kim WR, Harnois DM, Douglas DD, Burton J, Kulik LM (2012). Efficacy of neoadjuvant chemoradiation, followed by liver transplantation, for perihilar cholangiocarcinoma at 12 US centers. Gastroenterology.

